# Secure yet fragile: adversarial vulnerabilities of federated vision–language models in medical AI

**DOI:** 10.1038/s41598-026-48102-4

**Published:** 2026-04-16

**Authors:** Awal Ahmed Fime, Tasfia Zaman Samiha, Md Zarif Hossain, Saika Zaman, Ashfak Md Shibli, Abdur R Shahid, Zhen Ni, Ahmed Imteaj

**Affiliations:** 1 Department of Electrical Engineering and Computer Science, Florida Atlantic University, Boca Raton, FL 33431 USA; 2 Computer Science and Engineering, Khulna University of Engineering & Technology, Khulna, 9203 Bangladesh; 3 Athlete Den, Westbury, 11590 USA; 4 Computer Science, Southern Illinois University, Carbondale, IL 62901 USA

**Keywords:** Computational biology and bioinformatics, Mathematics and computing

## Abstract

Vision–Language Models (VLMs) enable powerful multimodal reasoning for medical image analysis, while federated learning allows collaborative training across institutions without sharing patient data. However, the adversarial robustness of federated medical VLMs remains largely unexplored. This work systematically evaluates the vulnerability of CLIP-based VLMs trained with four federated optimization strategies, FedAvg, FedProx, FedPer, and FedBN, on multiple medical datasets. We assess robustness under FGSM, PGD, BIM, and MI-FGSM attacks at varying strengths and show that client-level adversarial perturbations propagate through federated aggregation, causing severe accuracy degradation and high attack success rates, specially under iterative attacks. We further benchmark two training-free test-time defenses, Test-Time Counter-Attack (TTC) and CLIPure, and demonstrate that both mitigate adversarial effects, with CLIPure providing more consistent improvements across datasets and attack intensities. These results highlight fundamental robustness limitations of federated medical VLMs and underscore the need for effective defense mechanisms in distributed clinical deployments.

## Introduction

The fusion of vision and language through large-scale pre-training has redefined artificial intelligence, allowing Vision-Language Models (VLMs) to link visual perception with semantic understanding. In the medical domain, these models have shown in analyzing histopathological slides, retinal scans, and organ-level tissue images, offering an interpretable bridge between clinical visuals and diagnostic language^1,2^. However, as these models transition from controlled research settings to federated medical ecosystems, where multiple institutions collaboratively train and deploy them without sharing raw patient data, a critical concern emerges: *how robust are VLMs when operating under cyberattacks in such distributed environments?* Federated learning provides a promising paradigm for collaborative medical intelligence^3–6^, allowing multiple institutions to jointly train a global model while preserving patient privacy. Yet, this distributed framework introduces a subtle but profound vulnerability: adversarial perturbations can originate locally, propagate globally, and compromise model integrity system-wide^7,8^. Such perturbations, crafted to be visually imperceptible but semantically disruptive can corrupt local feature representations during training, infiltrate the global aggregation process, and distort downstream predictions. Within a clinical setting, this translates into grave consequences, such as the misclassification of pathological tissue or the misidentification of organ-level abnormalities, potentially undermining clinical reliability and patient safety^9^. Research on adversarial robustness has predominantly centered on centralized VLMs^10–12^, where both attacks and defenses can be explored under well-controlled experimental conditions. Some recent works^13–19^ focused on adversarial attacks to probe model susceptibility to imperceptible perturbations. Universal adversarial perturbations (UAPs) reveal a particularly concerning form of vulnerability in deep neural networks (DNNs), where a single input-agnostic manipulation can lead to incorrect predictions across a wide range of inputs. Prior work has shown that such perturbations can generalize across images and models, revealing vulnerabilities in DNNs. The authors^20^ proposed the concept of universal perturbations that can mislead classifiers across diverse samples using a single perturbation pattern. Some later studies further explored the generation of transferable universal perturbations using generative models^21^ and proposed a mechanism to learn universal perturbations from adversarial examples^22^. These studies have contributed valuable insights into the vulnerability of large multimodal systems; however, their scope has remained largely confined to general-purpose vision–language benchmarks rather than medical imaging. Only a limited number of works^23–28^ have begun to investigate how adversarial attacks affect medical datasets, where small perturbations to tissue structures, retinal layers, or organ contours can drastically alter diagnostic interpretations. Moreover, even these few studies typically examine isolated or centralized training regimes, overlooking the federated medical context in which real-world clinical data are inherently distributed and privacy-sensitive. Although recent architectures such as FARE^29^, TECOA^30^ have integrated defense mechanisms aimed at mitigating adversarial risk, their stability and reliability under federated medical conditions remain largely unexplored. This lack of systematic understanding underscores a fundamental gap in the current literature: how robust are medical VLMs when confronted with coordinated adversarial threats propagated across distributed, real-world datasets?

**Fig. 1:**
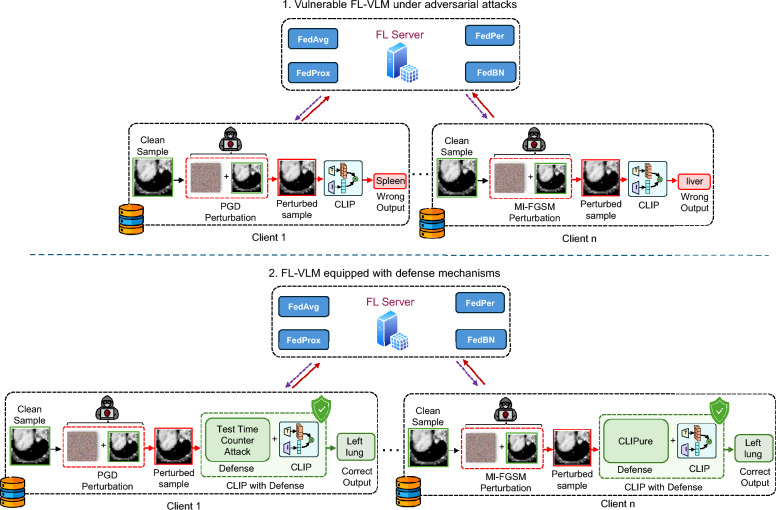
FL-VLM Adversarial Robustness Evaluation Pipeline. (Part 1) Standard federated VLM with four optimization algorithms (FedAvg, FedBN, FedPer, FedProx) fails under adversarial attacks (FGSM, PGD, BIM, MI-FGSM), producing misclassifications (“pleen” “iver” instead of correct organ identification (“eft Lung” shown in red). (Part 2) Same federated architecture enhanced with test-time defenses (Test-Time Counter-Attack and CLIPure purification) maintains correct classifications (green) under identical attacks. The comparison demonstrates fundamental vulnerability of undefended federated medical VLMs and effectiveness of training-free test-time defenses.

To address this challenge, we conduct a comprehensive investigation into the adversarial robustness of federated medical VLMs using the Three representative MedMNIST^31^ datasets: PathMNIST for histopathological tissue classification, OrganMNIST for organ-level structural analysis, and TissueMNIST for cellular-level differentiation. We examine how both conventional and robust encoders respond to gradient-based adversarial perturbations within the federated training process and quantify how robustness deteriorates as global updates integrate locally perturbed gradients. To further investigate the performance of existing defense mechanism for VLMs, we perform benchmarking of two recent defense mechanisms, Test-Time Counter-Attack (TTC) defense and CLPure defense under four different adversarial threats and three medical datasets (Fig. 1).

## Methods

We conduct our research in two sequential phases to examine the adversarial resilience of VLMs in federated medical environments. Phase 1 investigated how a standard VLM performs when collaboratively trained across distributed clients and subjected to different cyberattacks. Phase 2 extended this analysis to evaluate the robustness of two defense Test-Time Counter-Attack (TTC) and CLPure defense under identical adversarial conditions. Across both phases, three MedMNIST^31^ datasets are used to represent diverse medical imaging modalities and diagnostic tasks.

### Threat model and attacks

In our federated medical learning scenario, we assume a white-box adversary operating at the client level, an increasingly realistic threat in distributed hospital networks where local machines, imaging workstations, or telehealth endpoints may be compromised. The adversary has full access to the model architecture, weights, and input gradients on a single client but cannot modify global aggregation. Each client $k$ holds a dataset $D_k=\{(x_i,y_i)\}_{i=1}^{n_k},$ where $x_i\in \mathbb {R}^{H\times W\times 3}$ represents a medical image and $y_i$ its diagnostic label. Local training minimizes $\mathcal {L}_k(\theta )=\mathbb {E}_{(x,y)\sim D_k}\left[ \ell (f_\theta (x),y)\right] ,$ with $\ell$ denoting cross-entropy and $f_\theta (\cdot )$ the VLM classifier. The adversarial objective is to generate $x^{adv}$ such that $\Vert x^{adv}-x\Vert _p\le \epsilon , f_\theta (x^{adv})\ne y,$ where $p\in \{\infty ,2\}$. These perturbations remain visually imperceptible yet capable of distorting clinically relevant biomarkers in pathology, retinal OCT scans, organ CT slices, and renal tissue microscopy. Below, we describe each attack in detail, including how it impacts healthcare images and why it exposes vulnerabilities in VLM-based medical classifiers.

*FGSM: Fast gradient sign method (single-step linearization attack).*

FGSM^32^ is the most fundamental adversarial attack, leveraging the first-order Taylor approximation of the loss around input $x$. The attack assumes the loss increases most rapidly in the direction of the gradient $\nabla _x \ell (f_\theta (x),y)$. The perturbation is computed as $x^{adv}=x+\epsilon \cdot \textrm{sign}\!\left( \nabla _x\ell (f_\theta (x),y)\right) ,$ where $\epsilon$ is the magnitude of the perturbation. FGSM perturbs the input in the direction of maximal loss increase, and despite being a single-step attack, it is remarkably effective at corrupting fine-grained medical features. In PathMNIST, it can subtly warp glandular boundaries, crypt architectures, or nuclear staining cues that are critical for differentiating tissue types. In OrganAMNIST, it weakens structural organ contours or subtly alters CT intensity distributions, degrading anatomical consistency. In TissueMNIST, it distorts cellular morphology and cytoplasmic textures, disrupting the microscopic signatures on which the classifier relies. These perturbations are nearly invisible to clinicians but shift the VLM’s visual embedding, causing the language-aligned classifier to misinterpret semantic features like ’tumor’, ’retinal thinning’, or ’organ boundary’.

*PGD: Projected gradient descent (iterative first-order attack).*

**Fig. 2:**
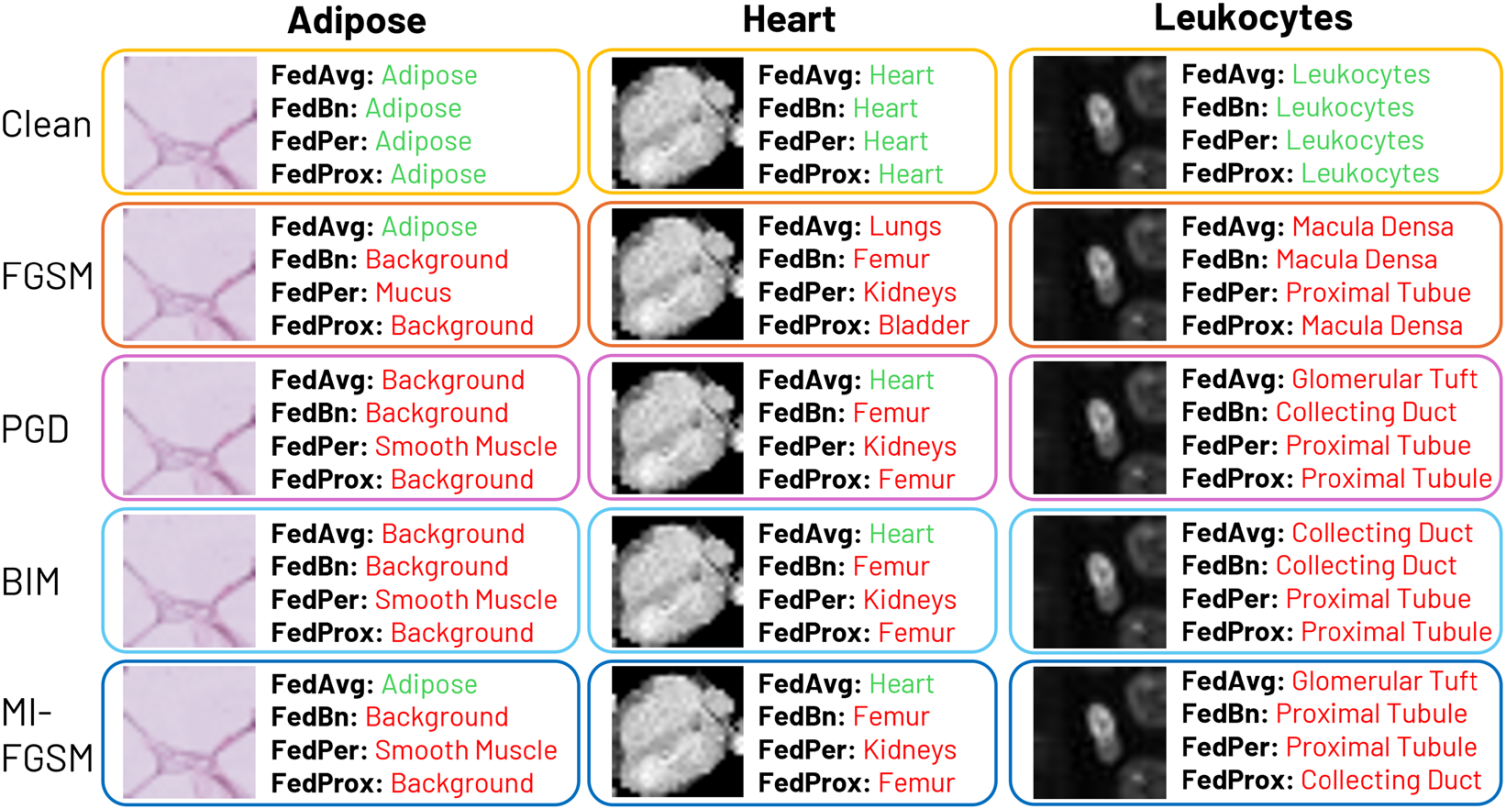
Qualitative examples of model responses for FedAvg, FedProx, FedPer, and FedBN on clean inputs and under FGSM, PGD, BIM, and MI-FGSM adversarial attacks. Each image corresponds to a class from OrganMNIST, PathMNIST, and TissueMNIST, highlighting the effect of adversarial perturbations on clean inputs.

PGD^33^ extends FGSM into an iterative procedure that repeatedly linearizes the loss and updates the input. At iteration $t$, the update rule is $x^{adv}_{t+1}=\Pi _{\mathcal {B}_\epsilon (x)}\!\left( x^{adv}_{t} + \alpha \cdot \textrm{sign}\!\left( \nabla _{x^{adv}_t}\ell (f_\theta (x^{adv}_t),y)\right) \right) ,$ where we set $\epsilon$ and step size $\alpha$ a predetermined value and run 10 iterations. The projection operator enforces the constraint $\Vert x^{adv}-x\Vert _\infty \le \epsilon ,$ ensuring the perturbation remains imperceptible. PGD performs iterative ascent on the loss surface, repeatedly adjusting for model non-linearity, which enables it to produce substantially more damaging perturbations than FGSM. In medical images, PGD can progressively erode malignant tissue boundaries in PathMNIST, inject structured noise into OrganAMNIST CT slices that resembles scanner artifacts, and alter intracellular contours or staining characteristics in TissueMNIST. These subtle yet systematic modifications shift the VLM’s visual embeddings away from clinically meaningful representations, leading to highly deceptive diagnostic errors. PGD is critical for federated robustness evaluation because it exploits small inconsistencies in each client’s data distribution. Because FL aggregates client updates, the model learns blurred or compromised feature representations. PGD reveals worst-case local vulnerabilities that traditional federated averaging method cannot correct (Fig. 2).

*BIM: Basic iterative method.* BIM^34^ extends FGSM into a multi-step attack that applies small, repeated perturbations in the direction of the gradient sign while clipping the result after each step. At iteration *t*, the update rule is $x^{adv}_{t+1} = \textrm{Clip}_{x,\epsilon }\!\left( x^{adv}_{t} + \alpha \cdot \textrm{sign}\!\left( \nabla _{x^{adv}_t}\ell (f_\theta (x^{adv}_t), y)\right) \right)$. The clipping operator enforces the constraint $\Vert x^{adv}-x\Vert _\infty \le \epsilon$, ensuring that each perturbation remains within an imperceptible bound around the original input. Unlike PGD, BIM does not introduce random initialization and therefore follows a deterministic trajectory along the local gradient landscape. This allows BIM to gradually sharpen adversarial artifacts that distort diagnostically relevant structures, such as blurring lesion margins in PathMNIST, amplifying low-contrast noise patterns in OrganMNIST CT slices that mimic acquisition irregularities, or subtly modifying cellular morphology and staining patterns in TissueMNIST. These progressive but constrained perturbations systematically shift the VLM’s internal representations away from semantically meaningful features, resulting in confident yet incorrect predictions. In federated settings, BIM is particularly effective at exposing cumulative vulnerabilities across rounds of training, as repeated small attacks can exploit minor client-specific biases and amplify representational drift that FL alone cannot fully mitigate, making BIM a valuable tool for assessing robustness under realistic, bounded adversarial conditions.

*MI-FGSM: Momentum iterative fast gradient sign method* MI-FGSM^35^ extends FGSM by incorporating a momentum term that accumulates gradient information across iterations to stabilize update directions and improve transferability. At iteration *t*, the gradient accumulator is updated as $g_{t+1} = \mu \cdot g_t + \frac{\nabla _{x^{adv}_t}\ell (f_\theta (x^{adv}_t), y)}{\Vert \nabla _{x^{adv}_t}\ell (f_\theta (x^{adv}_t), y)\Vert _1},$ and the adversarial example is updated via $x^{adv}_{t+1} = \textrm{Clip}_{x,\epsilon }\!\left( x^{adv}_{t} + \alpha \cdot \textrm{sign}(g_{t+1})\right) ,$ where $\mu$ is the momentum decay factor, $\epsilon$, step size $\alpha$, and 10 iterations are used. The momentum term suppresses oscillations in the gradient direction and encourages consistent traversal of the loss landscape, enabling MI-FGSM to escape poor local maxima and generate more transferable and structured perturbations than BIM or PGD. In medical imaging, this results in coherent adversarial patterns that propagate along anatomical or histological structures, such as shifting lesion boundaries in PathMNIST, injecting globally consistent scanner like artifacts into OrganAMNIST CT slices, or modifying cellular texture distributions in TissueMNIST. These spatially correlated perturbations induce stable shifts in the VLM’s visual embeddings, leading to confident but incorrect clinical predictions. In FL, MI-FGSM is particularly effective at revealing cross-client vulnerabilities, as momentum-driven perturbations exploit shared representational biases across clients, exposing robustness failures that are not apparent under single-step or locally constrained adversarial attacks.

### Federated learning framework and optimization strategies

We tailor a hub-and-spoke federated learning framework in which a global server coordinates parameter aggregation while five clients perform local training and communicate updates in each communication round. Training is conducted for $T=50$ rounds, each consisting of $E=5$ local epochs and a batch size of 32, using stochastic gradient descent with a learning rate of 0.003, momentum 0.9, and weight decay $5\times 10^{-4}$. To emulate realistic medical data heterogeneity, a balanced non-IID data partitioning strategy is employed, where each client is assigned a dominant class comprising the majority of its local samples. Within this framework, four federated optimization algorithms are evaluated. FedAvg^36^ aggregates all model parameters via weighted averaging to produce a single global model. FedProx^37^ extends FedAvg by introducing a proximal regularization term that constrains local updates to remain close to the global model, improving stability under heterogeneous data distributions. FedPer^38^ separates the model into shared and personalized components by freezing the CLIP backbone as a shared representation while training a client-specific classification head locally without aggregation. FedBN^39^ incorporates local batch normalization layers while aggregating the remaining parameters globally, allowing each client to retain normalization statistics aligned with its local data distribution and improving generalization under feature distribution shifts.

### Vision–language model integration

**Fig. 3:**
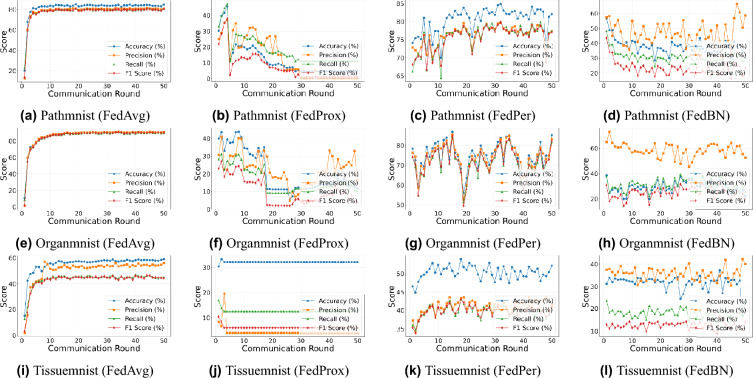
Performance evolution of accuracy, precision, recall, and F1-score across communication rounds for FedAvg, FedProx, FedPer, and FedBN on PathMNIST, OrganMNIST, and TissueMNIST.

We integrate a CLIP-based VLM as the core feature extractor, using the ViT-B/32 image encoder to obtain semantically rich visual embeddings. CLIP is pre-trained on 400M image–text pairs through a contrastive learning objective that aligns visual representations with corresponding natural-language descriptions. This large-scale multimodal training endows the encoder with strong generalization capabilities, enabling it to recognize complex visual patterns and map them into a shared semantic space where images and text coexist. In the medical context, this shared embedding space is particularly valuable because it allows the model to associate subtle diagnostic cues, such as glandular morphology in PathMNIST, organ boundaries in OrganAMNIST, and cellular features in TissueMNIST, with text-based class prompts corresponding to clinical terminology. This facilitates a form of “emantic grounding”that goes beyond purely image-driven representations and supports more interpretable classification pathways. To preserve this powerful pre-trained alignment, we keep the entire CLIP image encoder frozen during federated training. Freezing the encoder provides three key advantages. First, it prevents catastrophic forgetting of the multimodal relationships learned during pre-training, which are difficult to recover in small, domain-specific medical datasets. Second, it avoids representation drift caused by heterogeneous client data distributions, a common challenge in federated learning where institutions contribute non-identically distributed samples. If the encoder were allowed to update independently on each client, its semantic space would quickly diverge across sites, undermining global consistency. Third, freezing the encoder allows us to isolate and precisely measure the impact of adversarial perturbations on a stable shared embedding space. Because only the classifier head is updated during training, any adversarial vulnerability observed during evaluation reflects weaknesses in the encoder’s representational geometry rather than instability introduced by local fine-tuning. Building on this foundation, the classification process relies on CLIP’s language encoder to provide the semantic structure against which these embeddings are interpreted. Text prompts serve as class prototypes in the joint embedding space, and the ViT-B/32 image embedding is compared to these textual anchors using cosine similarity. This tight coupling between visual and linguistic representations ensures that classification decisions directly reflect the underlying vision–language semantics, enabling a clear analysis of how adversarial perturbations distort the alignment between medical images and their corresponding textual concepts in both centralized and federated settings (Fig. 3).

### Training environment, data preparation, and adversarial attack setup

All experiments are implemented using Python 3.11 and PyTorch 2.6.0 and are executed on an NVIDIA RTX A6000 GPU. Model checkpoints are saved at every communication round, preserving both server and client states, with an automatic resume mechanism to ensure training continuity. The experimental pipeline relies on OpenAI CLIP for vision–language modeling, the MedMNIST benchmark for medical imaging datasets, and scikit-learn for performance evaluation. Three datasets from the MedMNIST collection are used to cover diverse biomedical imaging modalities: PathMNIST for colorectal histopathology with nine classes, OrganAMNIST for organ-level CT analysis with eleven classes, and TissueMNIST for kidney cortex cell classification with eight classes, each provided with standard training, validation, and test splits to ensure reproducibility. All datasets are processed through a unified preprocessing pipeline to ensure compatibility with CLIP, where images are resized to $224\times 224$, grayscale inputs are expanded to three channels, and data augmentation is applied using random horizontal flips and random rotations within $\pm 15^\circ$. Pixel intensities are normalized using a mean and standard deviation of [0.5, 0.5, 0.5]. Adversarial robustness is evaluated under three attack strength settings. According to Table 1, strong attacks use $\epsilon = 0.05$ and $\alpha = 0.003$, medium attacks use $\epsilon = 0.03$ and $\alpha = 0.001$, and weak attacks use $\epsilon = 0.01$ and $\alpha = 0.0006$. Except for FGSM, which relies only on $\epsilon$ as a single-step perturbation, all other attacks incorporate both $\epsilon$ and step size $\alpha$ to generate iterative adversarial examples.

**Table 1:** SSIM-based perceptual quality analysis justifying the adversarial perturbation budget $\varepsilon$. Mean SSIM ${\uparrow }$ measures structural similarity between clean and perturbed images (SSIM $\ge 0.85$ = imperceptible for medical imaging). $\ell _\infty$ and $\ell _2$ norms quantify perturbation magnitude. $\checkmark$ = imperceptible; ✗ = perceptible. (Reviewer Point B).

Model	$\varepsilon$	OrganAMNIST				PathMNIST				TissueMNIST			
		SSIM$\,{\uparrow }$	$\ell _\infty$	$\ell _2$	Imperceptible	SSIM$\,{\uparrow }$	$\ell _\infty$	$\ell _2$	Imperceptible	SSIM$\,{\uparrow }$	$\ell _\infty$	$\ell _2$	Imperceptible
FedAvg	0.010	0.9834	0.0100	3.751	$\checkmark$	0.9823	0.0100	3.880	$\checkmark$	0.9763	0.0100	3.877	$\checkmark$
	0.030	0.8799	0.0300	11.241	$\checkmark$	0.8638	0.0300	11.640	$\checkmark$	0.8232	0.0300	11.561	✗
	0.050	0.7487	0.0500	18.713	✗	0.7037	0.0500	19.398	✗	0.6356	0.0500	18.983	✗
FedBN	0.010	0.9834	0.0100	3.751	$\checkmark$	0.9823	0.0100	3.880	$\checkmark$	0.9763	0.0100	3.877	$\checkmark$
	0.030	0.8798	0.0300	11.242	$\checkmark$	0.8637	0.0300	11.640	$\checkmark$	0.8232	0.0300	11.561	✗
	0.050	0.7486	0.0500	18.715	✗	0.7038	0.0500	19.398	✗	0.6356	0.0500	18.984	✗
FedPer	0.010	0.9834	0.0100	3.751	$\checkmark$	0.9823	0.0100	3.880	$\checkmark$	0.9763	0.0100	3.877	$\checkmark$
	0.030	0.8799	0.0300	11.241	$\checkmark$	0.8638	0.0300	11.640	$\checkmark$	0.8232	0.0300	11.561	✗
	0.050	0.7487	0.0500	18.713	✗	0.7038	0.0500	19.398	✗	0.6356	0.0500	18.984	✗
FedProx	0.010	0.9834	0.0100	3.751	$\checkmark$	0.9823	0.0100	3.880	$\checkmark$	0.9763	0.0100	3.877	$\checkmark$
	0.030	0.8798	0.0300	11.241	$\checkmark$	0.8638	0.0300	11.640	$\checkmark$	0.8231	0.0300	11.561	✗
	0.050	0.7486	0.0500	18.714	✗	0.7038	0.0500	19.398	✗	0.6355	0.0500	18.984	✗

###  CLIP with adversarial defenses

We leverage two test-time defense mechanisms to mitigate adversarial attacks on CLIP-based models. Since our setting focuses on fine-tuning CLIP on medical imaging datasets, test-time defenses are particularly suitable, as they do not require costly adversarial training (e.g., SLADE^40^, FARE^41^, Sim-CLIP^42^ ).

*Test-time Counter Attack* Test-Time Counter (TTC)^43^ is a training-free defense strategy that exploits the expressive power of CLIP’s pre-trained vision encoder to counteract adversarial perturbations during inference. TTC is motivated by the observation that adversarial examples generated by loss-maximizing attacks exhibit false stability, meaning their embeddings remain unusually stable under small random perturbations. TTC formulates defense as a counter optimization process at test time, where the image embedding produced by CLIP is treated as an anchor and a counter perturbation is iteratively computed to increase the distance between the embeddings of the original and perturbed images under a bounded perturbation budget. This optimization is carried out using a small number of projected gradient descent steps while keeping all model parameters fixed. To avoid degrading performance on clean samples, TTC tailors a thresholded weighted counterattack strategy that detects false stability based on embedding drift induced by random noise and applies counterattacks only when there is an attack.

*CLIPure* CLIPure^44^ is a test-time purification-based defense that improves adversarial robustness by operating directly in CLIP’s latent embedding space rather than in pixel space. The approach is motivated by the observation that CLIP’s vision-language aligned latent space is smoother and more semantically structured, which makes it more suitable for distinguishing adversarial samples from clean ones. Instead of explicitly removing perturbations in pixel space, CLIPure performs purification by iteratively adjusting the image embedding to maximize its likelihood under the clean embedding distribution. CLIPure estimates likelihood using a diffusion prior conditioned on CLIP text embeddings. During purification, embeddings are normalized to unit vectors to align with CLIP’s cosine similarity-based representation, and gradient ascent is applied to refine the embedding direction. Since CLIPure operates entirely at test time without modifying model parameters, it can defend against unseen attacks while maintaining strong clean image performance.

## Results

In our experiment, we quantify the performance using classification accuracy, macro-averaged precision, recall, and F1-score. Root Mean Square Error (RMSE) is used to measure the deviation between predicted and true outputs. For robustness analysis, the attack success rate (ASR) and the Robust Accuracy (Acc) are computed to capture the trade-off between predictive accuracy and vulnerability to adversarial manipulation.

### Performance comparison of pre-trained and fine-tuned CLIP models without attack

Table 2 summarizes the clean performance of CLIP-based models before and after federated fine-tuning across three medical datasets. The pre-trained CLIP model performs poorly on all tasks, achieving very low precision, recall, F1-score, and accuracy, which confirms that domain-specific adaptation is essential for medical image understanding. After federated fine-tuning, all methods show substantial improvements, but with notable differences across optimization strategies and datasets. FedAvg consistently achieves the strongest overall performance, particularly on OrganMNIST and PathMNIST, where it attains high accuracy and balanced precision–recall, indicating effective global knowledge aggregation. FedProx provides competitive and stable performance, especially on OrganMNIST, suggesting that constraining local updates helps mitigate client heterogeneity while preserving generalization. FedBN improves over the pre-trained baseline but lags behind FedAvg and FedProx, reflecting sensitivity to local normalization statistics in heterogeneous medical data. FedPer shows mixed behavior, performing well on PathMNIST but significantly underperforming on OrganMNIST, highlighting the tradeoff introduced by personalization in preserving global semantic consistency. The results demonstrate that federated fine-tuning is critical for achieving strong clean performance, while the choice of federated optimization method plays a key role in balancing accuracy, stability, and cross-dataset generalization.

**Table 2:** Performance comparison of the pre-trained CLIP model and its fine-tuned variants using FedAvg, FedPer, FedBN, and FedProx. Results are reported in terms of Precision (P), Recall (R), Accuracy (Acc), F1-score (F1), and Root Mean Square Error (RMSE) on the OrganMNIST, PathMNIST, and TissueMNIST datasets. Higher Precision, Recall, Accuracy, and F1-score (${\uparrow }$) and lower Root Mean Square Error (${\downarrow }$) indicate better performance.

	Model	Organmnist					Pathmnist					Tissuemnist				
		P ${\uparrow }$	R ${\uparrow }$	F1 ${\uparrow }$	Acc ${\uparrow }$	RMSE ${\downarrow }$	P ${\uparrow }$	R ${\uparrow }$	F1 ${\uparrow }$	Acc ${\uparrow }$	RMSE ${\downarrow }$	P ${\uparrow }$	R ${\uparrow }$	F1 ${\uparrow }$	Acc ${\uparrow }$	RMSE ${\downarrow }$
Pre trained	CLIP	4.14	4.47	0.45	4.47	0.28	1.39	11.73	2.48	11.73	0.31	13.02	23.83	16.38	23.83	0.31
Fine tuned	FedAvg	90.2	90.12	89.95	90.12	0.121	85.83	84.74	85.11	84.74	0.1561	59.26	58.96	56.59	58.96	0.2601
	FedBN	75.8	72.93	73.18	72.93	0.2199	74.99	72.88	72.26	72.88	0.2405	46.91	41.7	39.42	41.7	0.306
	FedPer	44.9	40.84	37.31	40.84	0.2662	77.86	75.75	73.94	75.75	0.2169	57.92	45.96	46.4	45.96	0.2926
	FedProx	78.28	76.08	76.38	76.08	0.1691	79.56	75.45	76.33	75.45	0.1935	43.72	45.99	37.08	45.99	0.2984

### Robustness of fine-tuned federated VLMs under adversarial attacks

Table 3 demonstrates that federated CLIP-based medical VLMs are highly vulnerable to adversarial perturbations, with performance degrading systematically as attack strength increases across FGSM, PGD, BIM, and MI-FGSM. Under weak FGSM attacks, FedAvg retains moderate accuracy on OrganMNIST but rapidly deteriorates on PathMNIST and TissueMNIST as attacks intensify, where ASR exceeds 70 consider percent and accuracy drops below 20 percent in strong settings. FedBN and FedPer exhibit consistently poor robustness even under weak attacks, often collapsing to near-random accuracy, indicating that localized normalization and personalization amplify adversarial sensitivity in distributed medical settings. Iterative attacks such as PGD and BIM are substantially more destructive, driving accuracy close to zero and ASR above 90 percent for most methods and datasets, revealing how small client-level perturbations propagate through federated aggregation and corrupt global semantic alignment. FedProx shows slightly improved stability relative to FedBN and FedPer, suggesting that constraining local updates mitigates but does not eliminate adversarial drift. Across datasets, OrganMNIST is comparatively more resilient, while TissueMNIST, which relies on fine-grained cellular morphology, is the most fragile under all attack types. Overall, the results highlight that strong clean performance does not translate to adversarial robustness and that existing federated optimization strategies fail to preserve the semantic integrity of vision–language representations under coordinated adversarial attacks.

**Table 3:** Performance of CLIP models under various adversarial attacks with weak, medium and strong settings. We report classification accuracy (Acc) and attack success rate (ASR) for FedAvg, FedPer, FedBN and FedProx under FGSM, PGD, BIM and MI-FGSM attacks across three medical datasets (OrganMNIST, PathMNIST, and TissueMNIST). Higher Acc (${\uparrow }$) and lower ASR (${\downarrow }$) indicate better robustness. The results highlight substantial performance degradation under stronger attacks and notable differences among federated optimization methods.

Attack	Model	Organmnist						Pathmnist						Tissuemnist					
		Weak		Medium		Strong		Weak		Medium		Strong		Weak		Medium		Strong	
		Acc ${\uparrow }$	ASR ${\downarrow }$	Acc ${\uparrow }$	ASR ${\downarrow }$	Acc ${\uparrow }$	ASR ${\downarrow }$	Acc ${\uparrow }$	ASR ${\downarrow }$	Acc ${\uparrow }$	ASR ${\downarrow }$	Acc ${\uparrow }$	ASR ${\downarrow }$	Acc ${\uparrow }$	ASR ${\downarrow }$	Acc ${\uparrow }$	ASR ${\downarrow }$	Acc ${\uparrow }$	ASR ${\downarrow }$
FGSM	FedAvg	66.34	24.31	56.25	34.69	53.69	37.45	28.47	58.43	19.76	70.61	18.91	76.8	5.79	63.73	7.61	71.5	16.1	70.31
	FedBN	20.24	67.49	15.98	72.74	18.46	71.63	15.77	70.95	9.0	80.4	6.77	84.15	7.24	77.32	8.28	81.1	7.1	80.82
	FedPer	6.97	56.77	6.6	61.8	18.46	71.63	15.46	51.95	14.78	54.07	12.81	58.22	15.46	65.67	16.54	67.64	16.54	67.2
	FedProx	31.31	56.0	27.96	60.77	29.51	61.14	22.16	44.67	17.03	50.28	29.69	60.27	13.48	63.23	11.95	74.09	10.0	79.7
PGD	FedAvg	77.01	13.64	64.38	26.39	49.07	42.07	37.84	48.72	20.25	67.03	12.42	76.48	3.73	64.91	0.36	70.21	0.0	72.78
	FedBN	2.77	92.33	0.03	96.88	0.0	97.13	1.45	94.29	0.01	96.66	0.0	96.82	0.03	91.74	0.0	91.84	0.02	91.28
	FedPer	0.84	77.12	0.04	85.16	0.0	97.13	0.13	84.82	0.0	85.65	0.01	89.0	0.01	74.89	0.0	76.15	0.01	79.77
	FedProx	5.63	86.66	0.11	94.72	0.01	94.34	4.97	88.19	0.22	95.56	0.0	94.56	0.11	69.42	0.04	72.39	0.21	71.05
BIM	FedAvg	74.19	16.48	62.82	27.96	48.68	42.5	31.98	54.69	19.26	67.99	12.65	76.43	2.27	66.86	0.24	70.44	0.0	72.87
	FedBN	3.67	90.78	0.61	95.95	0.01	97.38	1.21	95.1	0.14	96.39	0.03	97.65	0.03	92.25	0.02	92.16	0.02	93.05
	FedPer	1.51	75.52	0.24	82.17	0.01	97.38	0.17	85.08	0.03	86.57	0.0	91.96	0.02	74.73	0.01	76.7	0.01	78.13
	FedProx	7.3	84.5	1.76	92.2	0.0	93.08	5.92	86.67	1.73	93.38	0.0	93.04	0.09	70.07	0.18	72.	0.16	76.4
MI-FGSM	FedAvg	74.46	16.16	63.4	27.31	48.83	42.23	34.35	52.19	20.68	66.39	12.74	76.1	3.19	65.21	0.46	69.62	0.0	72.65
	FedBN	4.39	89.14	1.28	95.1	0.01	98.42	2.47	92.72	0.81	95.93	0.0	98.09	0.02	92.98	0.0	94.55	0.0	95.44
	FedPer	0.88	77.41	0.15	82.52	0.01	98.42	0.67	84.51	0.04	86.52	0.0	90.06	0.02	74.51	0.0	75.62	0.0	75.79
	FedProx	7.21	83.31	2.13	90.2	0.01	92.63	7.37	83.79	3.55	90.28	0.0	92.47	0.01	70.89	0.0	74.13	0.0	77.75

### Evaluation of adversarial robustness of federated VLMs with test-time counter-attack defense

We evaluate the effectiveness of the Test-Time Counter-Attack (TTC) defense in mitigating adversarial perturbations across different attack types, strengths, and federated optimization strategies in Tables 4 and 5. TTC consistently reduces attack success rates and partially recovers classification accuracy compared to the undefended setting, demonstrating its ability to counter adversarial effects without retraining. The benefits of TTC are most pronounced for FedAvg and FedProx, which maintain relatively stable accuracy across weak, medium, and strong attacks on OrganMNIST and PathMNIST, indicating that globally consistent representations benefit more from test-time correction. In contrast, FedBN and FedPer remain highly vulnerable under iterative attacks such as PGD and BIM, where ASR often exceeds 90 percent and accuracy remains low even with TTC, suggesting that local normalization and personalization limit the effectiveness of embedding-level counterattacks. Across datasets, OrganMNIST shows the greatest recovery under TTC, while TissueMNIST continues to exhibit higher ASR and lower accuracy, reflecting the inherent difficulty of defending fine-grained cellular morphology. In summary, TTC provides meaningful but uneven robustness gains, highlighting the need for stronger or complementary defenses medical settings.

**Table 4:** Performance of CLIP models under adversarial attacks with Test-Time Counter-Attack (TTC) defense (Organmnist & Pathmnist). Results are mean±std over three runs.

Attack	Model	Organmnist						Pathmnist					
		Weak		Medium		Strong		Weak		Medium		Strong	
		Acc ${\uparrow }$	ASR ${\downarrow }$	Acc ${\uparrow }$	ASR ${\downarrow }$	Acc ${\uparrow }$	ASR ${\downarrow }$	Acc ${\uparrow }$	ASR ${\downarrow }$	Acc ${\uparrow }$	ASR ${\downarrow }$	Acc ${\uparrow }$	ASR ${\downarrow }$
FGSM	FedAvg	20.2±0.41	21.26±0.63	17.22±0.54	28.44±0.72	15.91±0.69	30.41±0.81	28.15±0.44	53.97±0.77	24.85±0.46	64.37±0.88	24.37±0.62	70.68±0.93
	FedBN	3.79±0.94	67.56±1.21	4.22±0.98	72.28±1.32	4.71±1.02	71.96±1.29	19.78±0.75	69.92±1.34	15.01±0.81	77.67±1.41	14.64±0.83	81.24±1.47
	FedPer	6.19±0.72	40.82±1.02	7.01±0.76	45.6±1.05	7.48±0.81	47.78±1.09	17.84±0.65	4.71±0.33	17.37±0.63	3.89±0.31	17.1±0.61	4.47±0.34
	FedProx	8.46±0.81	54.04±1.11	9.48±0.84	57.64±1.14	11.31±0.89	57.56±1.16	16.89±0.67	4.3±0.35	16.73±0.66	4.75±0.36	16.62±0.65	4.76±0.37
PGD	FedAvg	20.97±0.44	13.56±0.53	20.0±0.48	24.15±0.66	16.19±0.59	34.54±0.81	31.34±0.47	47.41±0.73	26.41±0.49	62.53±0.88	22.74±0.46	70.11±0.93
	FedBN	3.19±0.91	92.75±1.33	2.6±0.87	97.46±1.44	1.43±0.82	97.6±1.45	16.3±0.72	94.55±1.41	17.3±0.74	96.94±1.46	12.23±0.66	96.89±1.44
	FedPer	8.02±0.74	77.46±1.18	5.71±0.66	85.23±1.27	4.41±0.61	88.0±1.31	19.43±0.69	84.16±1.21	6.7±0.58	85.99±1.26	1.88±0.44	89.65±1.34
	FedProx	6.04±0.69	87.6±1.23	4.2±0.63	94.83±1.37	2.69±0.58	94.58±1.36	17.55±0.68	88.7±1.24	8.29±0.62	94.86±1.35	1.59±0.46	96.82±1.41
BIM	FedAvg	20.75±0.45	16.18±0.54	19.87±0.47	25.19±0.66	15.69±0.52	34.93±0.81	29.86±0.46	52.58±0.74	25.82±0.48	63.2±0.87	22.7±0.45	69.99±0.92
	FedBN	3.23±0.92	91.28±1.32	2.28±0.88	96.66±1.41	1.34±0.81	97.86±1.45	15.97±0.72	94.94±1.40	13.62±0.69	96.46±1.43	12.01±0.66	97.62±1.46
	FedPer	7.77±0.74	76.35±1.16	6.64±0.71	82.26±1.23	3.54±0.59	89.87±1.33	16.3±0.66	84.75±1.22	9.37±0.63	86.27±1.25	1.55±0.43	91.5±1.36
	FedProx	5.78±0.68	85.17±1.21	3.96±0.63	92.52±1.33	3.08±0.61	92.97±1.34	17.88±0.69	87.03±1.24	16.1±0.67	94.23±1.36	1.25±0.41	96.99±1.42
MI-FGSM	FedAvg	20.77±0.46	15.94±0.53	19.88±0.48	24.81±0.65	15.75±0.53	34.81±0.80	30.08±0.47	50.53±0.72	25.96±0.49	62.28±0.86	22.34±0.44	69.83±0.91
	FedBN	2.31±0.89	89.33±1.29	1.84±0.86	95.65±1.40	1.34±0.82	98.6±1.46	18.31±0.73	92.9±1.36	15.13±0.69	95.86±1.41	11.85±0.66	97.97±1.46
	FedPer	6.01±0.71	77.91±1.18	5.11±0.66	83.0±1.24	3.09±0.59	89.67±1.32	7.1±0.52	84.16±1.21	2.53±0.47	86.23±1.25	0.42±0.33	90.0±1.34
	FedProx	3.81±0.64	85.52±1.22	3.13±0.61	91.56±1.33	2.26±0.57	92.19±1.34	20.99±0.71	83.58±1.18	15.07±0.68	90.7±1.31	1.02±0.38	96.63±1.41

**Table 5:** Performance of CLIP models under adversarial defense on tissuemnist. Results are mean±std over three runs.

Attack	Model	Test-time counter-attack defense						CLIPure defense					
		Weak		Medium		Strong		Weak		Medium		Strong	
		Acc ${\uparrow }$	ASR ${\downarrow }$	Acc ${\uparrow }$	ASR ${\downarrow }$	Acc ${\uparrow }$	ASR ${\downarrow }$	Acc ${\uparrow }$	ASR ${\downarrow }$	Acc ${\uparrow }$	ASR ${\downarrow }$	Acc ${\uparrow }$	ASR ${\downarrow }$
FGSM	FedAvg	32.07±0.35	63.65±0.71	32.07±0.37	71.35±0.91	32.07±0.39	70.23±0.88	5.81±0.33	63.65±0.72	7.68±0.36	71.45±0.91	16.12±0.44	70.28±0.89
	FedBN	8.36±0.69	77.58±1.39	7.4±0.64	80.38±1.44	7.69±0.66	80.64±1.46	5.65±0.61	76.92±1.41	9.40±0.64	78.75±1.43	11.46±0.66	77.72±1.42
	FedPer	22.99±0.74	64.62±1.12	22.52±0.72	62.63±1.08	23.44±0.77	66.92±1.14	10.66±0.62	60.19±0.96	12.81±0.64	65.30±1.01	14.40±0.67	69.49±1.05
	FedProx	13.17±0.59	55.45±1.04	17.2±0.64	53.13±1.01	11.15±0.56	77.25±1.18	14.31±0.59	53.09±0.94	20.76±0.64	49.76±0.91	22.99±0.67	49.76±0.92
PGD	FedAvg	32.07±0.36	64.88±0.74	32.07±0.38	70.2±0.89	32.07±0.39	72.74±0.95	3.72±0.28	64.86±0.74	0.35±0.21	70.20±0.88	0.00±0.20	72.78±0.92
	FedBN	9.39±0.62	92.43±1.37	9.33±0.61	92.2±1.36	9.33±0.62	91.35±1.33	0.10±0.30	90.77±1.29	0.02±0.28	89.81±1.26	0.03±0.29	88.67±1.23
	FedPer	23.98±0.77	74.95±1.09	29.0±0.83	76.26±1.11	25.48±0.79	80.06±1.16	0.10±0.29	75.54±1.08	0.07±0.27	76.54±1.09	0.13±0.31	79.33±1.12
	FedProx	8.47±0.59	69.08±1.02	8.49±0.58	72.27±1.05	22.7±0.76	70.88±1.03	0.43±0.34	66.95±0.98	0.19±0.32	69.65±1.01	0.50±0.35	67.63±0.99
BIM	FedAvg	32.07±0.36	66.85±0.76	32.07±0.37	70.38±0.89	32.07±0.38	72.86±0.95	2.25±0.26	66.83±0.76	0.25±0.21	70.39±0.89	0.00±0.20	72.84±0.93
	FedBN	9.44±0.62	93.04±1.37	9.39±0.61	92.64±1.36	9.33±0.62	93.46±1.38	0.12±0.29	90.47±1.28	0.06±0.28	90.96±1.29	0.04±0.28	90.88±1.28
	FedPer	24.02±0.77	74.68±1.09	24.03±0.76	77.11±1.12	25.24±0.79	78.32±1.15	0.09±0.28	74.89±1.07	0.11±0.29	76.27±1.09	0.15±0.30	76.86±1.10
	FedProx	8.67±0.59	69.92±1.03	8.35±0.58	72.62±1.06	8.11±0.57	76.55±1.11	0.54±0.35	67.00±0.99	0.46±0.34	69.99±1.02	0.58±0.36	71.72±1.04
MI-FGSM	FedAvg	32.07±0.36	65.19±0.75	32.07±0.37	69.56±0.88	32.07±0.38	72.65±0.94	3.17±0.27	65.19±0.74	0.47±0.23	69.58±0.88	0.00±0.20	72.62±0.92
	FedBN	9.43±0.62	93.62±1.38	9.37±0.61	95.04±1.40	9.31±0.60	95.7±1.42	0.07±0.28	91.57±1.30	0.01±0.26	92.50±1.32	0.00±0.25	91.43±1.30
	FedPer	24.07±0.77	74.36±1.09	24.26±0.76	75.77±1.11	26.26±0.79	75.54±1.10	0.07±0.27	73.42±1.05	0.05±0.26	74.22±1.06	0.04±0.26	76.69±1.09
	FedProx	8.47±0.59	70.57±1.04	8.04±0.57	73.91±1.08	11.59±0.64	77.63±1.12	0.08±0.28	67.50±1.00	0.07±0.28	69.99±1.02	0.06±0.28	72.35±1.05

**Table 6:** Performance of CLIP models with CLIPure defense under adversarial attacks (Organmnist & Pathmnist). Results are mean±std over three runs.

Attack	Model	Organmnist						Pathmnist					
		Weak		Medium		Strong		Weak		Medium		Strong	
		Acc ${\uparrow }$	ASR ${\downarrow }$	Acc ${\uparrow }$	ASR ${\downarrow }$	Acc ${\uparrow }$	ASR ${\downarrow }$	Acc ${\uparrow }$	ASR ${\downarrow }$	Acc ${\uparrow }$	ASR ${\downarrow }$	Acc ${\uparrow }$	ASR ${\downarrow }$
FGSM	FedAvg	66.12±0.58	21.22±0.64	59.35±0.53	28.50±0.74	57.67±0.55	30.57±0.79	32.95±0.47	53.94±0.76	26.06±0.49	64.35±0.87	24.99±0.46	70.74±0.94
	FedBN	19.35±0.83	67.71±1.22	15.72±0.79	73.06±1.34	16.91±0.81	74.40±1.37	18.62±0.76	68.29±1.29	17.31±0.74	71.13±1.32	16.82±0.73	71.95±1.34
	FedPer	13.24±0.71	91.98±1.26	16.79±0.75	93.14±1.29	16.19±0.73	92.89±1.27	9.39±0.54	78.51±1.08	11.63±0.57	81.63±1.11	11.60±0.56	82.02±1.13
	FedProx	34.25±0.77	58.88±1.05	27.49±0.69	65.54±1.12	28.00±0.71	67.02±1.17	26.45±0.66	67.69±1.19	23.12±0.63	71.41±1.23	22.03±0.61	74.65±1.27
PGD	FedAvg	73.75±0.61	13.53±0.48	63.32±0.55	24.19±0.66	53.18±0.52	35.12±0.79	39.03±0.49	47.47±0.73	24.62±0.46	62.65±0.86	19.23±0.42	69.93±0.91
	FedBN	2.45±0.86	92.59±1.33	0.01±0.72	96.88±1.43	0.00±0.70	97.02±1.44	1.73±0.65	95.77±1.41	0.00±0.62	97.31±1.45	0.00±0.60	96.82±1.42
	FedPer	28.18±0.73	90.59±1.24	34.32±0.77	93.08±1.28	38.67±0.81	94.57±1.31	24.62±0.69	81.95±1.11	40.74±0.84	84.28±1.15	48.50±0.89	91.25±1.24
	FedProx	4.51±0.63	88.10±1.21	0.02±0.51	94.68±1.34	0.00±0.48	94.44±1.32	3.94±0.59	89.78±1.23	0.07±0.50	95.52±1.37	0.00±0.47	97.38±1.42
BIM	FedAvg	71.15±0.59	16.18±0.55	62.40±0.56	25.17±0.68	52.95±0.53	35.46±0.80	34.05±0.47	52.62±0.75	24.05±0.45	63.22±0.86	19.39±0.43	70.07±0.92
	FedBN	3.03±0.87	91.14±1.30	0.37±0.75	96.18±1.42	0.00±0.70	97.46±1.45	1.53±0.63	95.74±1.41	0.22±0.59	97.59±1.45	0.03±0.55	97.88±1.47
	FedPer	28.10±0.72	90.48±1.23	30.64±0.75	91.93±1.26	36.46±0.80	93.95±1.30	25.49±0.70	81.74±1.10	32.92±0.78	84.29±1.14	44.29±0.87	90.33±1.22
	FedProx	5.70±0.66	85.84±1.19	1.03±0.57	92.79±1.31	0.01±0.49	92.43±1.29	5.28±0.62	88.09±1.21	1.23±0.58	94.42±1.34	0.00±0.47	96.87±1.41
MI-FGSM	FedAvg	71.35±0.60	15.94±0.54	62.71±0.57	24.79±0.67	52.97±0.54	35.32±0.79	35.97±0.48	50.58±0.74	24.82±0.46	62.24±0.85	18.94±0.42	69.93±0.91
	FedBN	4.11±0.88	88.63±1.27	0.84±0.79	95.08±1.38	0.00±0.70	97.47±1.45	2.56±0.66	93.93±1.34	0.85±0.60	96.64±1.41	0.00±0.55	98.09±1.48
	FedPer	32.47±0.78	91.42±1.25	35.68±0.81	92.24±1.27	39.28±0.85	94.08±1.31	29.12±0.73	81.62±1.09	37.51±0.82	84.42±1.15	49.78±0.90	89.30±1.20
	FedProx	4.75±0.67	86.40±1.20	0.73±0.56	91.68±1.32	0.00±0.48	91.84±1.31	7.52±0.64	84.16±1.18	2.65±0.60	91.21±1.29	0.01±0.49	96.59±1.40

### Adversarial robustness of federated VLMs with CLIPure defense

To this end, we tailor the CLIPure defense against the same adversarial attacks for the medical datasets across the four Federated Learning algorithms. In Tables 6 and 5, we demonstrate that CLIPure consistently improves the robustness of federated CLIP-based models against adversarial attacks by reducing attack success rates while preserving or partially recovering classification accuracy across datasets and attack strengths. Compared to the undefended setting, CLIPure yields noticeable gains for FedAvg and FedProx, particularly under FGSM and moderate PGD and BIM attacks on OrganMNIST and PathMNIST, indicating that purification in the CLIP embedding space effectively restores semantic alignment disrupted by adversarial perturbations. In contrast, FedBN and FedPer remain more vulnerable under strong iterative attacks, where ASR often remains high and accuracy improvements are limited, suggesting that local normalization and personalized heads reduce the consistency needed for effective embedding purification. Across datasets, OrganMNIST again shows the strongest recovery, while TissueMNIST continues to be the most challenging, with only modest accuracy gains under strong attacks due to its reliance on fine-grained cellular features. The results indicate that CLIPure provides a stronger and more consistent defense than TTC, particularly for globally aligned federated models, while still highlighting the limits of test-time defenses under severe adversarial conditions.

### Backdoor poisoning attack

The results in Table 7 show that several FL methods are highly vulnerable under this stronger threat model. For example, FedAvg exhibits very high ASR on PathMNIST and TissueMNIST (up to 99.93% and 100.00%, respectively), indicating severe susceptibility to trigger-based corruption. FedBN and FedProx provide partial resistance on some datasets, but their ASR remains substantial, while FedPer shows mixed behavior, achieving 0.00% ASR on OrganAMNIST but remaining highly vulnerable on TissueMNIST. These findings clarify that the security risk in federated medical learning is not limited to ephemeral evasion at inference time, compromised clients can also introduce persistent malicious behavior into the global model through poisoning. We have revised the threat model discussion accordingly to better distinguish these two attack surfaces.

**Table 7:** Backdoor poisoning attack evaluation across FL models and datasets. Clean Acc (%) ${\uparrow }$ = accuracy on clean test set after poisoning (higher = stealthier); ASR (%) ${\downarrow }$ = Attack Success Rate on triggered inputs (lower = more robust).

Attack	Model	OrganAMNIST						PathMNIST						TissueMNIST					
		Weak		Medium		Strong		Weak		Medium		Strong		Weak		Medium		Strong	
		Acc$\,{\uparrow }$	ASR$\,{\downarrow }$	Acc$\,{\uparrow }$	ASR$\,{\downarrow }$	Acc$\,{\uparrow }$	ASR$\,{\downarrow }$	Acc$\,{\uparrow }$	ASR$\,{\downarrow }$	Acc$\,{\uparrow }$	ASR$\,{\downarrow }$	Acc$\,{\uparrow }$	ASR$\,{\downarrow }$	Acc$\,{\uparrow }$	ASR$\,{\downarrow }$	Acc$\,{\uparrow }$	ASR$\,{\downarrow }$	Acc$\,{\uparrow }$	ASR$\,{\downarrow }$
Backdoor	FedAvg	33.24	70.78	33.24	70.78	33.24	70.78	18.69	99.93	18.69	99.93	18.69	99.93	32.07	100.00	32.07	100.00	32.07	100.00
	FedBN	44.72	49.85	45.26	59.16	44.72	47.88	39.53	49.09	37.28	65.82	41.46	61.02	41.15	99.55	41.93	96.96	41.32	99.65
	FedPer	40.62	0.00	35.75	0.00	32.68	0.00	82.17	9.40	82.88	11.40	83.01	11.42	32.07	100.00	32.07	100.00	32.07	100.00
	FedProx	61.28	50.20	65.34	41.00	60.69	45.66	76.00	50.65	76.27	55.99	76.11	53.59	44.96	99.84	43.48	99.89	43.79	99.98

### Computational overhead of test-time defense

Although both TTC and CLIPure operate as test-time defenses, their computational costs differ substantially. TTC requires only a small number of gradient-based adaptation steps during inference ($N=2$), resulting in relatively low overhead. In contrast, CLIPure includes an additional iterative purification stage, making it considerably more expensive at test time. Empirically, TTC increases inference latency by only about $1.2\times$ over the standard CLIP model, whereas CLIPure incurs an overhead of approximately $1.8\times$. These results suggest that TTC is the more practical and deployment-friendly defense, especially for latency-sensitive medical applications where efficiency is critical.

### Full fine-tuned CLIP under adversarial attacks

**Table 8:** Adversarial attack results on OrganAMNIST across attack levels on full-finetuned CLIP model.

Attack	Model	Weak		Mid		Strong		Attack	Model	Weak		Mid		Strong	
		Acc ${\uparrow }$	ASR ${\downarrow }$	Acc ${\uparrow }$	ASR ${\downarrow }$	Acc ${\uparrow }$	ASR ${\downarrow }$			Acc ${\uparrow }$	ASR ${\downarrow }$	Acc ${\uparrow }$	ASR ${\downarrow }$	Acc ${\uparrow }$	ASR ${\downarrow }$
FGSM	FedAvg	5.12	10.82	4.66	18.56	4.44	23.82	BIM	FedAvg	5.15	9.73	4.66	14.41	1.96	33.38
	FedBN	95.54	4.15	92.33	7.55	88.91	11.02		FedBN	96.29	3.04	95.57	4.15	87.87	12.16
	FedPer	88.05	5.91	84.05	10.30	81.58	13.13		FedPer	91.44	4.60	89.49	6.63	81.85	14.40
	FedProx	88.82	6.40	84.67	10.74	82.27	13.16		FedProx	89.23	6.70	86.49	9.47	75.01	21.06
PGD	FedAvg	5.21	8.79	4.71	13.79	2.36	31.12	MI-FGSM	FedAvg	5.21	9.04	4.86	12.91	3.05	28.56
	FedBN	96.41	2.76	95.62	4.09	89.33	10.52		FedBN	96.21	3.16	95.42	4.38	87.50	12.45
	FedPer	91.70	4.31	89.71	6.40	82.07	14.21		FedPer	91.06	4.97	89.04	7.09	80.73	15.52
	FedProx	89.36	6.62	86.47	9.51	74.77	21.43		FedProx	88.89	7.09	86.09	9.84	75.67	20.36

Table 8 reports the robustness of fully fine-tuned CLIP models under multiple adversarial attacks (FGSM, PGD, BIM, and MI-FGSM) across varying perturbation strengths. Overall, FedBN consistently achieves the best performance, maintaining high accuracy (above 87% even under strong attacks) while keeping ASR low, demonstrating strong robustness. In contrast, FedAvg performs poorly across all settings, with accuracy dropping to nearly random levels under stronger attacks. FedPer and FedProx show moderate robustness, with noticeable degradation in accuracy and increased ASR as attack strength increases. Across all methods, stronger attacks consistently reduce accuracy and increase ASR, highlighting the vulnerability of federated CLIP models to adversarial perturbations, while emphasizing the effectiveness of normalization-based personalization (FedBN) in improving robustness.

Full fine-tuning of the CLIP model is approximately 4.2$\times$ slower than training only the classification head and incurs significantly higher memory overhead due to the need to store gradients across the entire network. Moreover, given the limited size of typical medical datasets, full fine-tuning tends to degrade the generalization capability of the pretrained CLIP encoder, leading to a loss of its inherent cross-modal utility. This reduction in representational versatility restricts the model’s applicability to downstream tasks such as disease description generation and clinical decision support. To preserve the robustness and extensibility of the pretrained representations, we therefore keep the CLIP encoder frozen and optimize only the task-specific head.

## Discussion

This paper provides a critical analysis of the adversarial robustness of federated CLIP-based VLMs in medical imaging scenarios and reveals several important insights. First, the results consistently show that strong clean performance does not imply robustness under adversarial conditions. Although federated fine-tuning substantially improves accuracy over the pre-trained CLIP baseline, all evaluated federated optimization strategies remain highly vulnerable when adversarial perturbations are introduced at the client level. This behavior highlights how adversarial perturbations generated locally can propagate through the federated aggregation process and corrupt the global semantic alignment between visual embeddings and language prompts. The comparative analysis of federated optimization methods further reveals that algorithmic design choices significantly influence robustness. Dataset characteristics also play a critical role in adversarial susceptibility. OrganMNIST is comparatively more resilient across most settings, whereas PathMNIST and specially TissueMNIST exhibit substantially higher vulnerability. The fragility of TissueMNIST suggests that tasks relying on fine-grained cellular morphology are particularly sensitive to imperceptible perturbations, as small distortions can significantly alter the visual features that drive semantic alignment in VLMs. The evaluation of test-time defenses demonstrates that training-free mitigation strategies can partially alleviate adversarial effects but do not fully resolve the problem. Test-Time Counter-Attack reduces attack success rates and recovers some accuracy, particularly for globally consistent models such as FedAvg and FedProx, but its effectiveness diminishes under strong iterative attacks. CLIPure provides more consistent improvements across datasets and attack strengths by operating directly in the CLIP embedding space and restoring semantic alignment. However, CLIPure struggles under severe adversarial conditions, specially for TissueMNIST and for federated strategies that introduce representation inconsistency.

## Data Availability

All datasets used in this study are publicly available. OrganMNIST, PathMNIST, and TissueMNIST are part of the MedMNIST benchmark collection and can be accessed through the official MedMNIST repository https://medmnist.com/ and GitHub repository at https://github.com/MedMNIST/MedMNIST. The datasets are also archived and released under the Creative Commons Attribution 4.0 International (CC BY 4.0) license. Full details of the dataset construction and characteristics are described in the original publication (https://doi.org/10.1109/ISBI48211.2021.9434062).
